# Fraxinellone Has Anticancer Activity by Inducing Osteosarcoma Cell Apoptosis via Promoting Excessive Autophagy Flux

**DOI:** 10.3389/fphar.2021.653212

**Published:** 2021-04-19

**Authors:** Bin He, Wenkan Zhang, Jiaming He

**Affiliations:** ^1^ Department of Orthopedic Surgery, The Second Affiliated Hospital, Zhejiang University School of Medicine, Hangzhou, China; ^2^ The Second Affiliated Hospital, Zhejiang University School of medcine, Hangzhou, China; ^3^ Key Laboratory of Motor System Disease Research and Precision Therapy of Zhejiang Province, Hangzhou, China; ^4^ The Fourth Affiliated Hospital, Zhejiang University School of Medicine, Hangzhou, China

**Keywords:** fraxinellone, proliferation, cancer therapy, autophagy, apoptosis

## Abstract

Osteosarcoma is a malignant bone tumor that is easy to metastasize in the early stage and has a very poor prognosis. Fraxinellone (FRA) is one of the main components isolated from the *D. dasycarpus* plant. Its anti-inflammatory and neuroprotective effects have been confirmed, but the research on the anti-cancer effect of FRA and its potential mechanism is relatively scarce. In this study, we found that FRA inhibited the proliferation and migration of osteosarcoma cells HOS and MG63 in a dose-dependent manner. Immunofluorescence, fluorescence staining and western blotting analysis showed that FRA could simultaneously induce osteosarcoma cell apoptosis and increase autophagy flux. Subsequent turnaround experiments suggested that the pro-apoptotic effect of FRA was achieved through excessive autophagy flux. The results of the xenograft orthotopic model further supported the anti-cancer effects of FRA, indicating that FRA treatment inhibited the growth of osteosarcoma, and the pro-apoptotic and autophagy effects of FRA were also proved *in vivo*. These studies provide new ideas for the future treatment of osteosarcoma and offer theoretical support for the anti-cancer mechanism of FRA.

## Introduction

Osteosarcoma is one of the most common primary bone malignancies that occurs in children and adolescents, even though its incidence is very low, about three per million (Ritter and Bielack, 2010; Anderson, 2016). The pathological feature is the presence of malignant mesenchymal cells that produce osteoid and immature bone, and metastasize to a distant place in the early stage of the disease. The most common metastatic organ is the lung (Lin et al., 2017; Tang et al., 2019). The 5-years survival rate of non-metastatic patients is about 60%, while the 5-years survival rate of metastatic patients is only 20–30% (Biazzo and De Paolis, 2016; Harrison et al., 2018). Micrometastasis in the lungs that existed before the surgical resection of the primary tumor is the most important factor for the poor prognosis of those metastatic patients (Moore and Luu, 2014). At present, surgical treatment, neoadjuvant chemotherapy and postoperative adjuvant chemotherapy have improved the survival and prognosis of patients, but the survival rate of patients with osteosarcoma has not fundamentally changed, which has prompted us to develop novel treatment strategies (Isakoff et al., 2015).

*Dictamnus dasycarpus* is a traditional herbal medicine used to treat inflammatory diseases and has been used in Eastern countries such as Korea and China for thousands of years (Gu et al., 2011). According to recent studies, fraxinellone (FRA) is a limonin component isolated from *Dictamnus dasycarpus*. Compared with other ingredients, it is the main active ingredient with anti-inflammatory and neuroprotective affects (Jeong et al., 2010; Jung et al., 2018; Kim et al., 2019). In addition, studies have suggested that FRA has vasodilator activity (Kim et al., 2009). It is worth noting that the study found that FRA can down-regulate the STAT3 and HIF-1α signaling pathways and inhibit the expression of PD-L1, thereby inhibiting cancer cell proliferation and reducing tumor angiogenesis (Xing et al., 2018). However, research on the anti-tumor effect of FRA is very rare. Considering that traditional chinese medicine has shown good potential in clinical treatment in recent years, we want to explore whether it has anti-tumor activity against osteosarcoma.

A recent study revealed that FRA inhibits senescence via restoring the H_2_O_2_-impaired autophagic flux (Han et al., 2018). Autophagy, also known as type II cell death, is a process in which cells use lysosomes to degrade their damaged organelles and macromolecular substances under the control of autophagy-related genes (ATG) (Ravanan et al., 2017). Simply put, after the cell receives the autophagy-inducing signal, it forms a phagophore, and the phagophore continues to extend to absorb the components in the cytoplasm to form a spherical autophagosome. After the autophagosome is formed, it can fuse with the lysosome to become an autolysosome. After the inner membrane of the autophagosome is degraded by the lysosomal enzyme, the “cargo” in the autophagosome is also degraded, and the available product is transported to the cytoplasm. The residue is either discharged outside the cell or retained in the cytoplasm (Amaravadi et al., 2016; Yan et al., 2019). Autophagy plays an important role in cell metabolism, structural reconstruction, growth and development (Saha et al., 2018). The relationship between autophagy and tumors is very complicated and has not yet been fully elucidated. On the one hand, the enhancement of normal cell autophagy can exhibit the function of inhibiting tumorigenesis; on the other hand, tumor cells can also enhance cell autophagy to resist the stress response induced by hypoxia, metabolites, and therapeutic drugs (Onorati et al., 2018; Russo and Russo, 2018). In our current research, we have explored the regulatory effect of FRA on autophagy.

Cell death is a complex process regulated by the body. Apoptosis, as the first programmed cell death (PCD) program recognized, its role and regulatory network are relatively clear (Kaczanowski, 2016). In some cases, autophagy can inhibit apoptosis and is a survival way for cells to protect themselves. However, autophagy itself can also induce cell death, or it can be used as a backup mechanism to induce cell death in the absence of apoptosis. The two pathways are interrelated and regulate each other (Mariño et al., 2014). Research and use of these interactions will help to further reveal the occurrence and development of tumors and other diseases.

In this study, we found that FRA inhibited the proliferation and migration of the two cell lines of osteosarcoma HOS and MG63. In addition, it could induce tumor cell apoptosis by promoting autophagy of osteosarcoma cells, thereby inhibiting tumor growth *in vivo* and *in vitro*.

## Materials and Methods

### Cell Culture and Reagents

The human osteosarcoma cell lines MNNG/HOS (HOS) (CRL-1547TM, ATCC) and MG63 (CRL-1427TM, ATCC) were purchased from the Cell Bank of the Chinese Academy of Sciences (Shanghai, China). HOS and MG63 cells were cultured in Dulbecco’s modified Eagle’s medium (DMEM) with penicillin (100 units/ml)-streptomycin (100 units/ml) and 10% fetal bovine serum (FBS). The cells were incubated at 37°C in an environment with 1% O_2_, 94% N_2_, and 5% CO2 at 37°C. FRA (Cat: 28808-62-0) were purchased from Sigma-Aldrich technology (St. Louis, MO, United States); The purity of FRA was >95% in HPLC analysis.

### Cell Proliferation Assays

The inhibitory effect of FRA on osteosarcoma cells was tested by CCK8 kit (Dojindo Laboratories, Kumamoto, Japan). The cells were seeded into 96-well plates, each with about 4,000 cells. After the cells adhered to the bottom of the well, we changed the medium and cultured osteosarcoma cells with various concentrations of FRA(0–320 μM) for different periods of time (0–48 h). In order to verify the potential mechanism of FRA on autophagy, cells were treated with different concentrations of FRA and incubated with 3-MA (3 mM) or rapamycin (100 nM). Then, CCK8 reagent (10 μl) was added to each well, and after incubating for 2 h, the absorbance was measured at 450 nm using a MR7000 microplate reader (Dynatech, NV, United States). We evaluated cell proliferation viability through colony formation experiments. The cells in the exponential growth phase were seeded into a 6-well plate and allowed to adhere, and the cell density per well was controlled to 500 cells. The cells were treated with different concentrations of FRA(0, 40, and 80 μM) for 15 days. The colony was washed three times with 1x phosphate buffered saline (PBS), then fixed with paraformaldehyde for about 20 min, and stained with 0.1% crystal violet (Beyotime); Next, they were counted under a microscope and photographed. Count the number of colonies (>50 cells/colony).

### Cell Migration Analysis

A wound healing assay was used to detect the migration ability of two human osteosarcoma cell lines. When the cell proliferation in the 6-well plate reaches about 90% confluence, use a 10 μL pipette tip to evenly scrape the cell layer to form a scratch. After that, gently wash three times with PBS to remove scraped cell debris. After adding serum-free medium, immediately photograph the thickness of the scratch under the microscope. Then, culture with different conditions (control group, 40 μM FRA group, 80 μM FRA group). After 12 h of treatment, pictures of the cell scratches were taken again, and the wound area was analyzed using ImageJ software.

In addition, the classic transwell chamber (membrane with 8 μm pore size, Corning Life Sciences, United States) was used to further evaluate the migration ability of tumor cells. Add 600 μL treatment medium (control group, 40 μM FRA group, 80 μM FRA group) containing 10% FBS to the lower chamber. 10^6^ cells were resuspended in 200 µL of serum-free medium and then planted in the upper chamber. After incubating for 12 h, the cells were washed with PBS, then fixed with 4% paraformaldehyde for 30 min, and finally stained with 0.1% crystal violet for 10 min. Then, the cells in the upper layer of the chamber were wiped off with a cotton swab, the cells under the membrane were counted and photographed through an inverted microscope.

### Transmission Electron Microscope

Transmission electron microscopy (TEM) is a commonly used technique to observe the ultrastructure of cells (Harris, 2015). Through TEM, apoptotic bodies, nuclear condensation and autophagosomes can be clearly observed. Simply put, after the treated cells are fixed with 2.5% glutaraldehyde and 1% osmium acid, they are dehydrated with different concentrations of alcohol (30–100%), and then the cells are embedded in Epon to prepare ultrathin sections (70–90 nm) for observation under a transmission electron microscope.

### Cell Apoptosis Assays

Cell apoptosis was detected by flow cytometry and cells were stained using the reagent Annexin V-FITC/PI (Multi-Sciences, Hangzhou, Zhejiang, China). Cells were treated with different concentrations of FRA(0, 40 and 80 μM) and 3-MA (3 mM) or rapamycin (100 nM) for 24 h. After washing with 1XPBS two times, cells were resuspended in binding buffer. One hundred microliters of cell suspension were incubated with 5 µl annexin V FITC and 10 µl PI for 30 min at normal temperature in the dark. Cells were then detected by a flow cytometer (FACSCalibur, BD, San Jose, CA, United States).

### Hoechst 33342 Stain

The morphological changes of apoptotic cells can be observed by Hoechst 33342 staining, such as nuclear shrinkage and nuclear lysis. After washed with PBS, the treated cells were incubated with Hoechst 33342 dye solution in the dark for about 15 min. Then, the morphological changes of apoptotic cells were observed under a wavelength of 365 nm by a fluorescence microscope (Olympus, Tokyo, Japan).

### Western Blotting

Collect all the cells that have undergone different treatments, and then wash them twice with PBS. According to the instructions, the cells were lyzed with RIPA buffer (Sigma-Aldrich, St. Louis, Missouri, United States) containing a mixture of protease inhibitors and phosphatase inhibitors. After the protein supernatant is obtained, the protein concentration is measured by BCA protein assay kit (Beyotime). The same amount of protein samples was electrophoresed by 8–15% SDS-PAGE at 80 V, and then the proteins were transferred to a polyvinylidene fluoride (PVDF) membrane (Millipore, Billerica, MA, United States) in a humid environment. The membrane was blocked with 5% nonfat milk (BSA; Sigma-Aldrich) and incubated with the primary antibody at 4°C overnight. Wash with TBST 3 times, after 10 min each time, then each membrane was incubated with anti-rabbit or anti-mouse IgG sheep antibody (Huabio, Hangzhou, Zhejiang, China) for 1 h at room temperature. Visualize the reactive protein using an enhanced chemiluminescence kit (Millipore). The details for the primary antibodies are as follows: Bcl-2 (1:2000, Cat: ab182858, ABCAM, MA, United States), Cleaved-caspase-3 (1:1000, Cat: 9664S, CST), Cleaved-caspase-7 (1:1000, Cat: 8438S, CST), Autophagy Antibody Sampler Kit (1:1000, Cat: 4445T, CST), Phospho-SQSTM1/p62 (1:1000, Cat: 16177S, CST). The secondary antibodies were as follows: goat anti-rabbit IgG–HRP (1:2000, Cat: 7074S, CST) and goat anti-mouse IgG-HRP (1:2000, Cat: 7076S, CST).

### MDC Staining

The cells with different treatments were collected, and washed twice by wash buffer, then counted and adjusted their concentration to 10^6^/ml. An appropriate amount of 90 μl of cell suspension and 10 μl of MDC stain were move into a new EP tube, and mixed gently, incubated at room temperature for 30 min in the dark. Then washing the cells twice with wash buffer and discarding the supernatant. Add 100 μl of collection buffer to resuspend the cells, drop them onto a glass slide and add a cover glass. The cells were observed under a fluorescence microscope (excitation filter wavelength 355 nm, blocking filter wavelength 512 nm), counted and taken pictures.

### Turnaround Experiment

In order to verify that FRA indeed induces an increase in apoptosis by promoting autophagy of tumor cells, we incubated FRA-treated HOS and MG63 cells with autophagy inducer rapamycin (100 nM) or autophagy inhibitor 3-MA (3 mM). After incubating with rapamycin or 3-MA, the total protein is extracted.

The cytotoxicity in the turnover experiment was analyzed by CCK8 assay and flow cytometry. HOS and MG63 cells were seeded in 96-well plates (5 × 10^3^ cells/well) and six-well plates (1 × 10^5^ cells/well), respectively, and treated with the above conditions. Then conduct CCK8 test and flow cytometry to detect the rescue and aggravating effects of autophagy inhibitors or promoters.

### Immunofluorescence

Visually observe the expression intensity of related proteins in cells through immunofluorescence technology. The cells were treated with different concentrations of FRA (0, 80 μM) for 24 h. Following the conventional method, the treated cells were fixed with 4% paraformaldehyde, permeabilized with 0.5% Triton X-100, blocked with 5% BSA, and incubated with the primary antibody overnight at 4°C. After the secondary antibody was incubated for 1 h, the cells were observed and photographed under a fluorescence microscope.

### *In Vivo* Xenograft Assay

All experiments were carried out in accordance with the guidelines of the Ethics Committee of Zhejiang University and approved by the Research Ethics Committee of the Second Affiliated Hospital of Zhejiang University School of Medicine, China. All surgical procedures and applications on animals comply with IACUC guidelines. About 2 × 10^6^ stably transfected HOS cells [which have been transfected with luciferase (HOS-Luc) for *in vivo* imaging] were injected into the bone marrow cavity of the right tibia of a four-week-old nude mouse (Shanghai Experimental Animal Center, Chinese Academy of Sciences) (Sampson et al., 2013). After 7 days, those mice with too high or too low fluorescence intensity were eliminated, and then the remaining mice were randomly divided into three groups (three mice in each group). Starting from the seventh day, the FRA low-dose group received 50 mg/kg FRA per day by gavage, while the FRA high-dose group was given 100 mg/kg, and the mice in the control group received 200 uL PBS per day. After 21 days of treatment, the luminescence intensity of each mouse was measured and recorded by *in vivo* fluorescence. The mice were anesthetized with chloral hydrate, and all mice were sacrificed by cervical dislocation. Then, the tibial tumor was resected, weighed, and fixed with 4% paraformaldehyde for subsequent histological analysis.

### *In Vivo* Bioluminescence Assay

After intraperitoneal injection of 200 μL of fluorescein (100 mg/ml), the tumor-bearing mice were anesthetized with isoflurane inhalation. IVIS 200 imaging system was used for *in vivo* imaging, and Living Image Software (version 3.0.4, Xenogen, Hopkinton, MA, United States) was used to analyze the results.

### Histology and Immunohistochemistry

The tissue fixed in 4% formaldehyde was decalcified, embedded in paraffin, and cut into 4 μM sections. The tumor tissue and other organ tissue sections were deparaffinized in xylene solution and stained with hematoxylin and eosin. According to the instructions of the IHC kit (Boster Bio, China), the tumor tissue sections were deparaffinized, rehydrated and immunostained. Then, sections were extracted in 0.01 M pH 6.0 citrate buffer using heat-induced epitopes. After the sections were washed with PBS and blocked with BSA, they were incubated with the primary antibody (cleaved-caspase 3, LC3B, P62) at 4°C overnight. Then, the sections were incubated with the secondary antibody (Huabio, China) for 1 h at room temperature, and then stained with the enzyme substrate 3′,3-diaminobenzidinetetrahy-drochloride (DAB, Huabio, China). The staining intensity of each section was scored by Image Pro Plus 6.0. Each part was independently analyzed by two pathologists.

After deparaffinization and hydration, the sections were incubated with proteinase K (20 μg/ml) at 37°C for 30 min. Then the sections were blocked with 3% BAS and incubated with the primary antibody for 1 h at room temperature. Finally, the sections were incubated with the TUNEL reaction mixture, and after counterstaining, the sections were dehydrated and sealed, and then observed and photographed under a fluorescence microscope.

### Statistical Analysis

The results were expressed as the mean ± standard deviation (SD). A comparison of the results was performed with one-way ANOVA and Student’s t-test. All statistics were analyzed by IBM SPSS Statistics 20.0 software (IBM, Armonk, NY, United States). Statistically significant differences between groups were defined as *p* < 0.05.

## Results

### FRA Inhibits the Proliferation of HOS and MG63 Cells *In Vitro*

**FIGURE 1:**
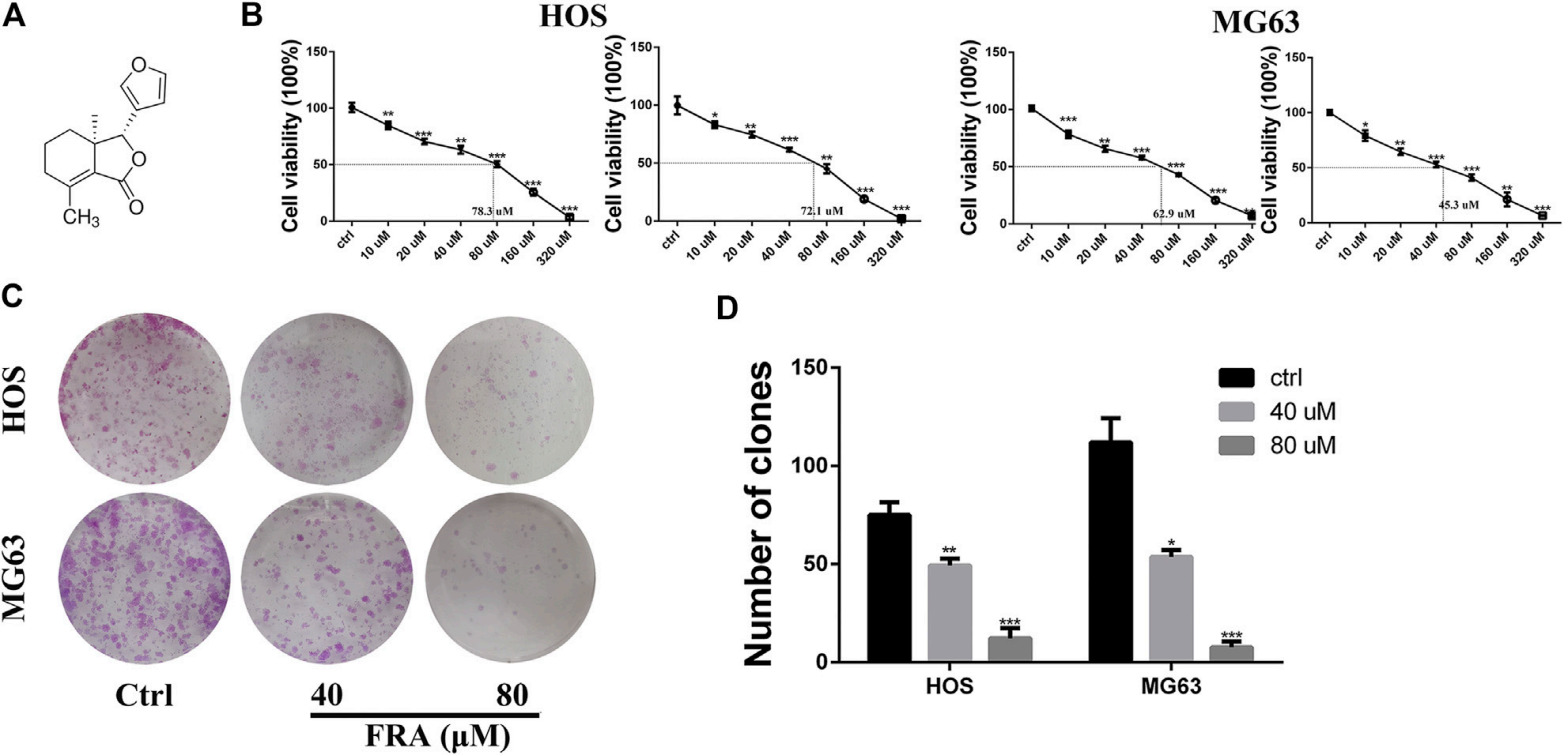
FRA inhibited osteosarcoma cell proliferation *in vitro*. **(A)** Chemical structure of fraxinellone (FRA). **(B)** Human osteosarcoma cell lines (HOS & MG63) were treated with different concentrations of FRA for 24 and 48 h before CCK8 assays were utilized. **(C)** Compared with control group, FRA significantly suppresses colony formation in HOS and MG63 cell lines. **(D)** Number of clones in plates were analyzed. These data were detected by a microplate reader and a microscope, respectively. *p < 0.05, **p < 0.01, and ***p < 0.001 compared with the controls.

One of the main effects of FRA on human osteosarcoma cells HOS and MG63 is to inhibit cell proliferation. The CCK8 assay and colony formation assay were used to evaluate cell viability (Figure 1). The results (Figure 1B) showed that as the dose of FRA increased, the viability of tumor cells would be more and more significantly inhibited. According to the numerical analysis of absorbance, the IC50 values of FRA in HOS cells is 78.3 μM (24 h) and 72.1 μM (48 h); while the IC50 values in MG63 cells are 62.9 μM (24 h) and 45.3 μM (48 h). In addition, as shown in Figure 1C, the results of colony formation assay showed that FRA can inhibit the monoclonal proliferation of HOS and MG63 cells to form clumps. After 15 days, there were about 450 ± 23 clonal clumps in the control group, 279 ± 12 in the 40 μM FRA group and 63 ± 5 in the 80 μM group (Figure 1D). The above results suggest that with the increase of FRA dose, the proliferation activity of the two types of osteosarcoma cells was significantly inhibited.

### FRA Inhibits the Migration Ability of HOS and MG63 Cells *in Vitro*

**FIGURE 2:**
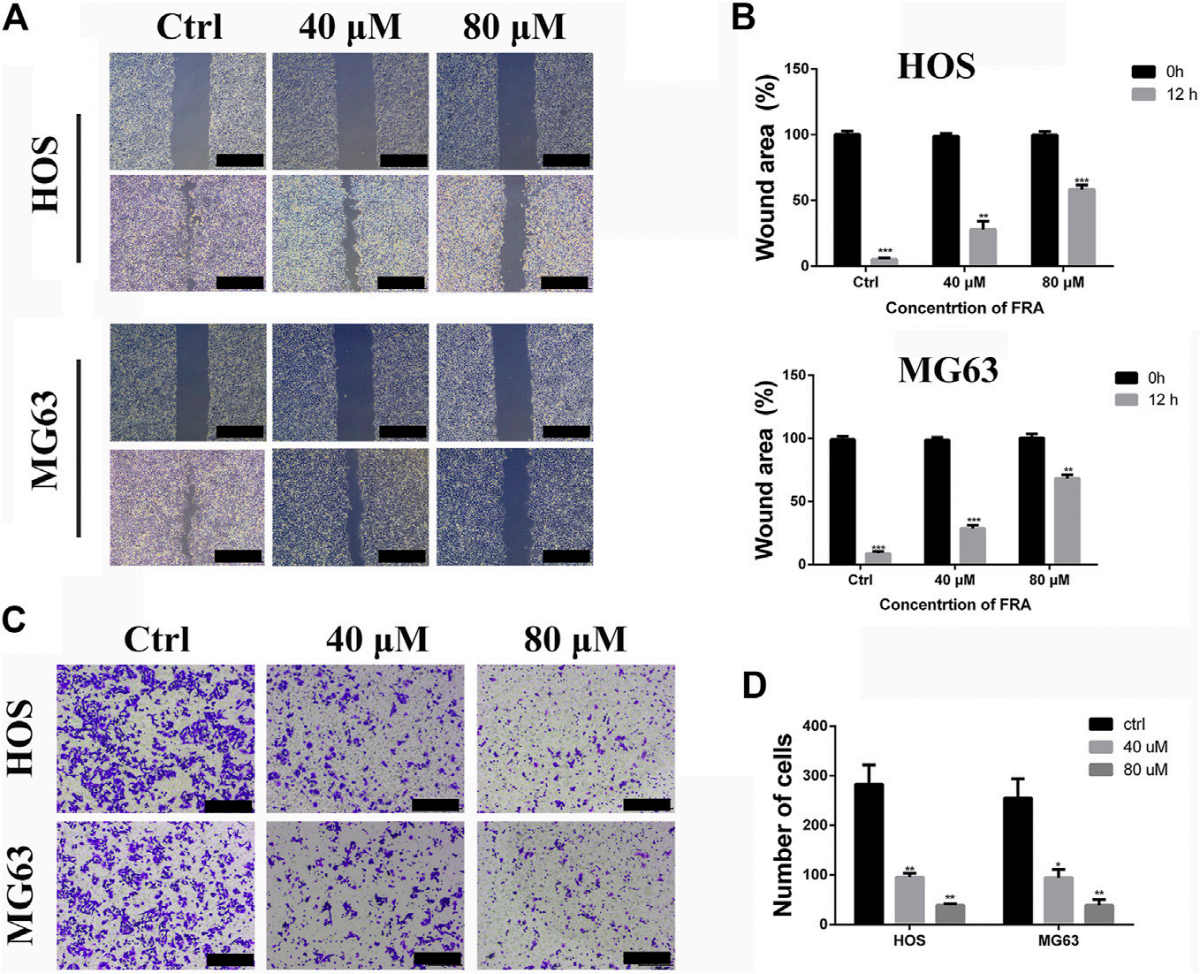
FRA inhibits osteosarcoma cell migration *in vitro*. **(A)** The HOS and MG63 osteosarcoma cell lines were incubated with various doses of FRA (0; 40 μM; 80 μM) for 12 h, and photographs taken at 0 and 12 h were statistically analyzed by Image J software. The results of wound-healing assay showed that FRA significantly blocked the cell recolonization in the wound area in the HOS and MG63 cell lines. **(C)** In the transwell assay under the same conditions, the number of cells in of the FRA treatment group was significantly lower than that of the control group, and the number of cells in the high dose was less than that in the low dose. Percentages of wound area **(B)** and number of clones **(D)** in the lower membrane were counted. Error bar = mean ± SEM of at least triplicate experiments. Magnification, ×40 **(A)**, ×100 **(C)**. Scale bar, 500 μm **(A)**, 200 μm **(C)**. *p < 0.05, **p < 0.01, ***p < 0.001, #*p* < 0.0001.

Based on the results obtained before, we selected low-dose concentration of 40 μM and high-dose concentration of 80 μM FRA for follow-up studies, and performed the cell wound healing assay at 12 h. In Figure 2A, compared with the control group, FRA significantly blocked the cell recolonization in the wound area in the HOS and MG63 cell lines. As shown in Figure 2B, the presence of FRA resulted in a significant reduction in the number of cells covered by the scratched area, and the inhibitory effect of the high-dose group was more obvious. In addition, we have also obtained consistent phenomena through the transwell assay. Relatives to the control, FRA could reduce the migration of these two types of osteosarcoma cells to the lower membrane of the chamber (Figures 2C,D). These data indicated that FRA inhibited the migration of human osteosarcoma cells HOS and MG63 in a dose-dependent manner.

### FRA Regulates Osteosarcoma Cell Autophagy and Apoptosis

**FIGURE 3:**
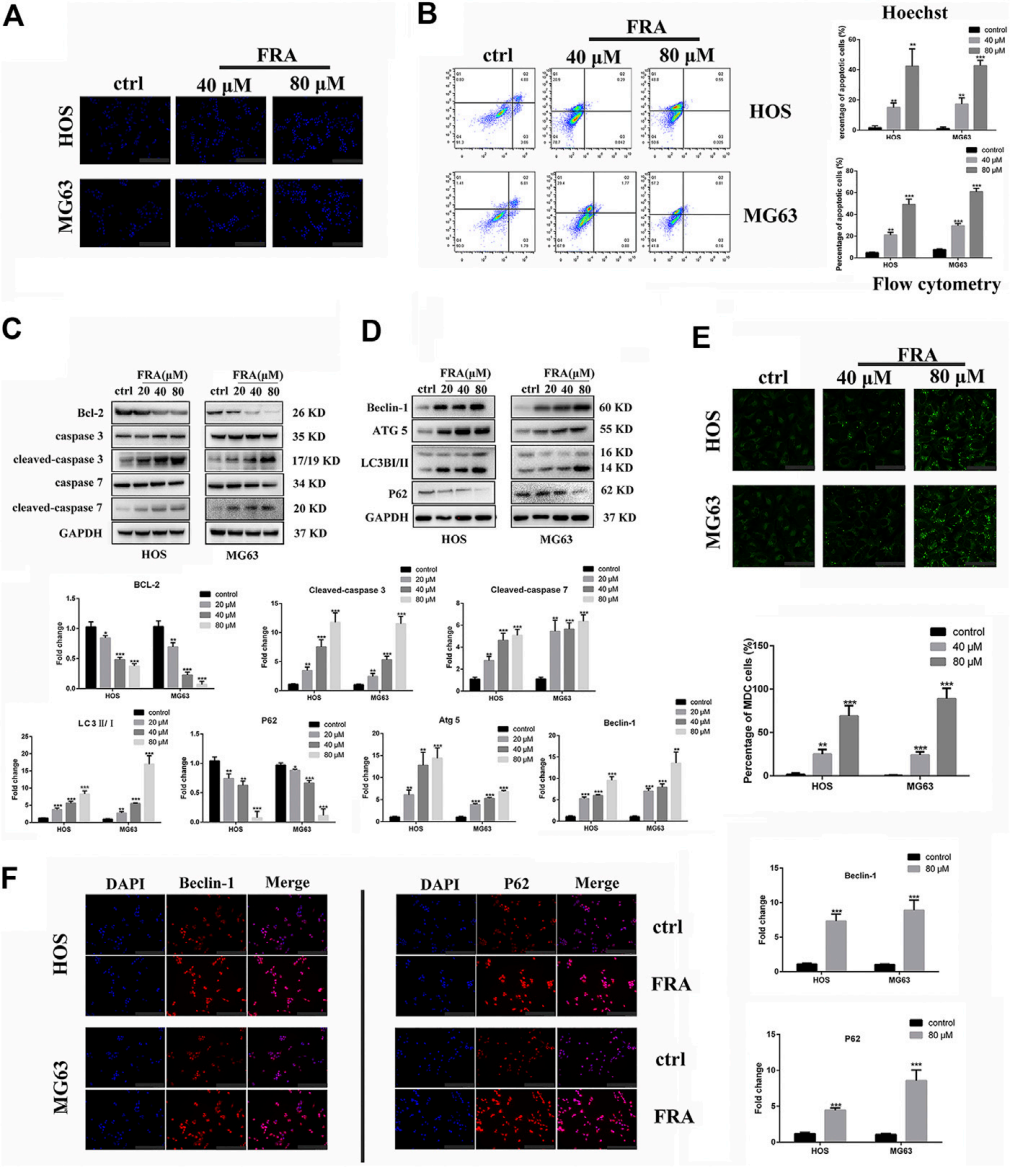
FRA induces autophagy and apoptosis of osteosarcoma cells *in vitro*. **(A)** Cells treated with different conditions were stained with Hoechst 33342, and results showed that the percentage of apoptotic cells in the FRA group was higher than in the control group. The highlight dots mean apoptotic cells. **(B)** After staining with an Annexin FITC/PI kit, the treated cells were tested by flow cytometry. The results indicated that the percent of total apoptotic cells significantly increased in the FRA-treated group in a dose manner. **(C)** The expression of the following apoptosis-related proteins was determined by Western blot analysis: Bcl-2,cleaved-caspase 3, caspase-3, cleaved−caspase 7, and caspase 7. **(D)** The expression of those autophagy-related proteins: Beclin-1, ATG5, LC3B and P62. **(E)** Cells treated with different conditions were stained with MDC, and results showed that the number of autophagosome in the FRA group was higher than in the control group. The green dots mean autophagosome. **(F)** The HOS and MG63 osteosarcoma cell lines were incubated with FRA (80 μM) or control for 24 h. The expression level of the Beclin-1 and P62 were significantly increased after FRA treatment detected via immunofluorescence assays. Each assay was repeated three times. Magnification, ×200 **(A)**, ×1000 **(D)**, ×200 **(F)**. Scale bar, 100 μm **(A)**, 20 μm **(E)**, 100 μm **(F)**. Error bar = mean ± SD of at least triplicate experiments. *p < 0.05, **p < 0.01, ***p < 0.001 versus control.

**FIGURE 4:**
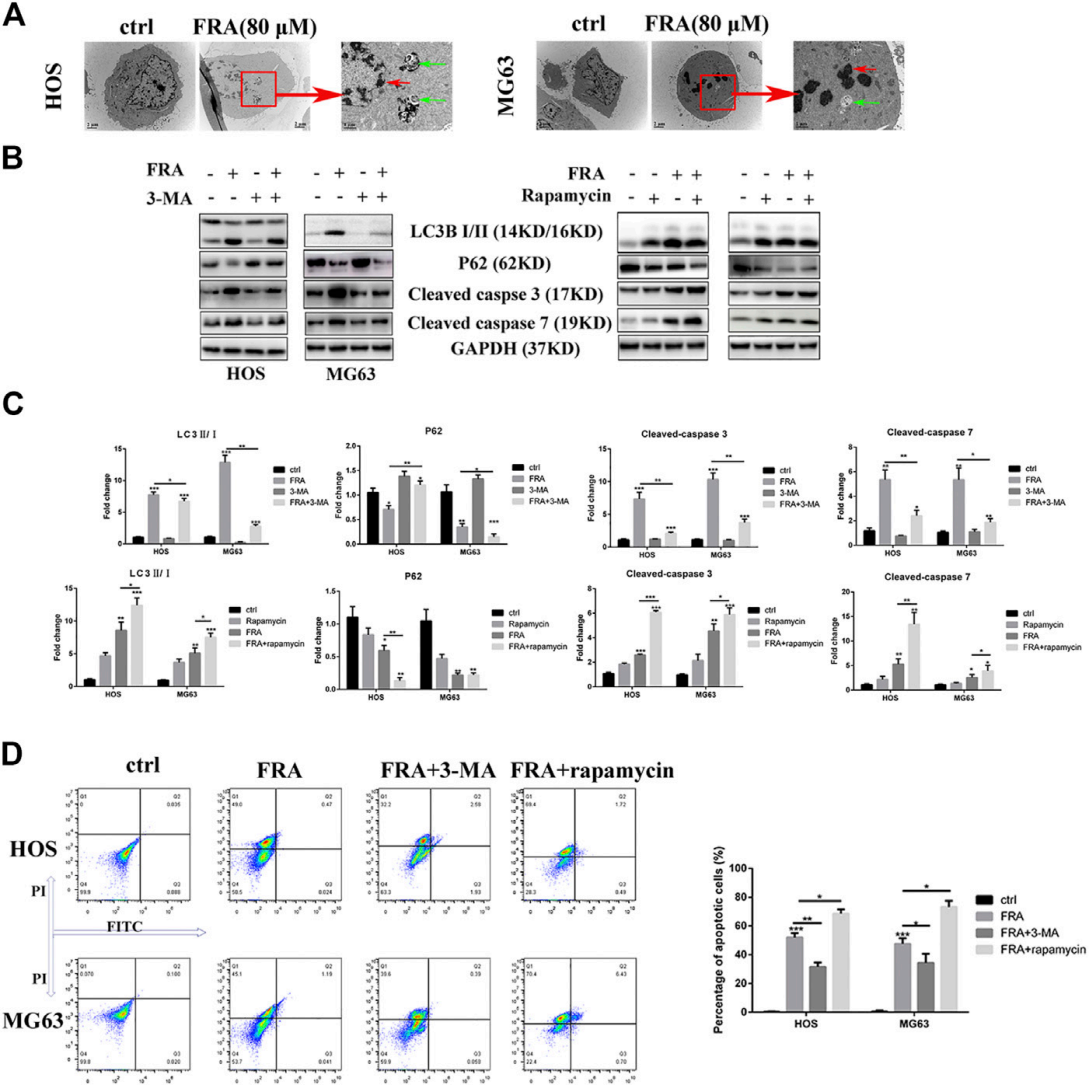
FRA induces cell apoptosis by promoting excessive autophagy flux. **(A)** Transmission electron microscopy (TEM) shows the morphological changes in cells induced treated with FRA. The red arrows mean apoptotic bodies and nuclear condensation. The green arrows mean autophagosomes. Magnification, ×10,000, Scale bar, 2 μm. HOS and MG63 cells were treated with or without FRA (8 μM), with or without 3-MA (3 mM), or with or without rapamycin (100 nM) for 24 h. **(B)** The protein expressions of LC3B, p62, cleaved-caspase 3 and cleaved-caspase 7 were measured by Western blotting. **(C)** The corresponding histogram statistics of these proteins. Each assay was repeated three times. **(D)**. The cells after different treatments were stained with the Annexin FITC/PI kit, and then the percentage of apoptotic cells was detected by flow cytometry. Error bar = mean ± SD. *p < 0.05, **p < 0.01, ***p < 0.001 versus control.

In addition to inhibiting cell migration, high concentrations of FRA can also induce apoptosis and autophagy. According to previous studies on FRA, the induction of apoptosis is its main anti-tumor effect. Therefore, we performed apoptosis-related assays in two cell lines to evaluate its specific apoptosis effects. In Figure 3A, the simple Hoechst 33342 staining results suggested that FRA could promote the generation of apoptotic cells with nuclear shrinkage and highlight. Annexin V-FITC/PI flow cytometry assay further confirmed that FRA induced apoptosis of HOS and MG63 cells. In terms of specific data in Figure 3B, the apoptosis rate of FRA treatment group (HOS: 40 μM 21.19%, 80 μM 48.55%; MG63: 40 μM 31.17%, 80 μM 60.01%) was significantly higher than Control group (HOS: NC 5.68%; MG63: NC 8.22%). The expression of apoptosis-related proteins was examined by western blotting to verify the pro-apoptotic effect induced by FRA. As shown in the Figure 3C, whether in the HOS or MG63 cell line, after FRA treatment, the protein expression of cleaved-caspase 3 and cleaved-caspase 7 protein were significantly up-regulated, while the Bcl-2 protein expression was down-regulated. Finally, as one of the gold standards for apoptosis detection, we performed TEM observations and photographed those FRA-treated cells. The pictures under the microscope in Figure 4A indicated that compared with the control group, the FRA group could clearly see the shrinkage of nuclear chromosomes (red arrows). These results all indicate that FRA induces apoptosis of HOS and MG63 osteosarcoma cell lines *in vitro.*

What is interesting is that we found in the TEM images that there were typical autophagosomes (green arrows) in the cytoplasm of tumor cells after FRA treatment. Considering the complex relationship between autophagy and apoptosis, we subsequently explored the influence of FRA on the autophagy of osteosarcoma cells. MDC is a specific eosinophilic stain commonly used to detect autophagosome formation. In Figure 3E, the results of MDC staining revealed that there were more autophagosomes in the FRA treatment group (green dots in the cytoplasm). In addition, the higher the concentration of FRA, the more dots in the cytoplasm and the higher the fluorescence intensity. Western blot and immunofluorescence were used to evaluate the autophagy regulation of FRA. As shown in Figure 4D, relative to the control group, after cells were treated with FRA, the ratio of LC3B-II/LC3B-I bands and the expression of ATG5 and beclin-1 protein increased significantly in a dose-dependent manner. The current study believes that P62 is a substrate involved in the degradation of autophagosomes (Moscat et al., 2016). In our research results, the expression of P62 protein will also increase with the appearance of FRA. Immunofluorescence staining also directly showed that the expression of Beclin-1 and P62 in FRA-treated cells increased. These results suggest that FRA can promote the formation of autophagosomes and accelerate its degradation, thereby increasing the autophagy flux of cells.

### FRA Induces Cell Apoptosis by Promoting Excessive Autophagy Flux

Then we explore the specific relationship between FRA-induced autophagy and apoptosis. Through the intervention of 100 nM rapamycin (autophagy promoter) and 3 mM 3-MA (autophagy inhibitor), we found that FRA promoted the apoptosis process of osteosarcoma cells by inducing autophagy flux. Results of western blot experiment showed that with the addition of rapamycin, FRA could further increase the apoptosis-related proteins, and flow cytometry detected more apoptotic cells. After adding the autophagy blocker 3-MA, the pro-apoptotic effect of FRA was obviously inhibited (Figures 4B,D). In Figure 4C, the expression of these proteins that shown in figure B were analyzed. The above experimental results verified our hypothesis that FRA caused apoptosis through excessive autophagy flux.

### FRA Hinders Tumor Growth *In Vivo*

**FIGURE 5:**
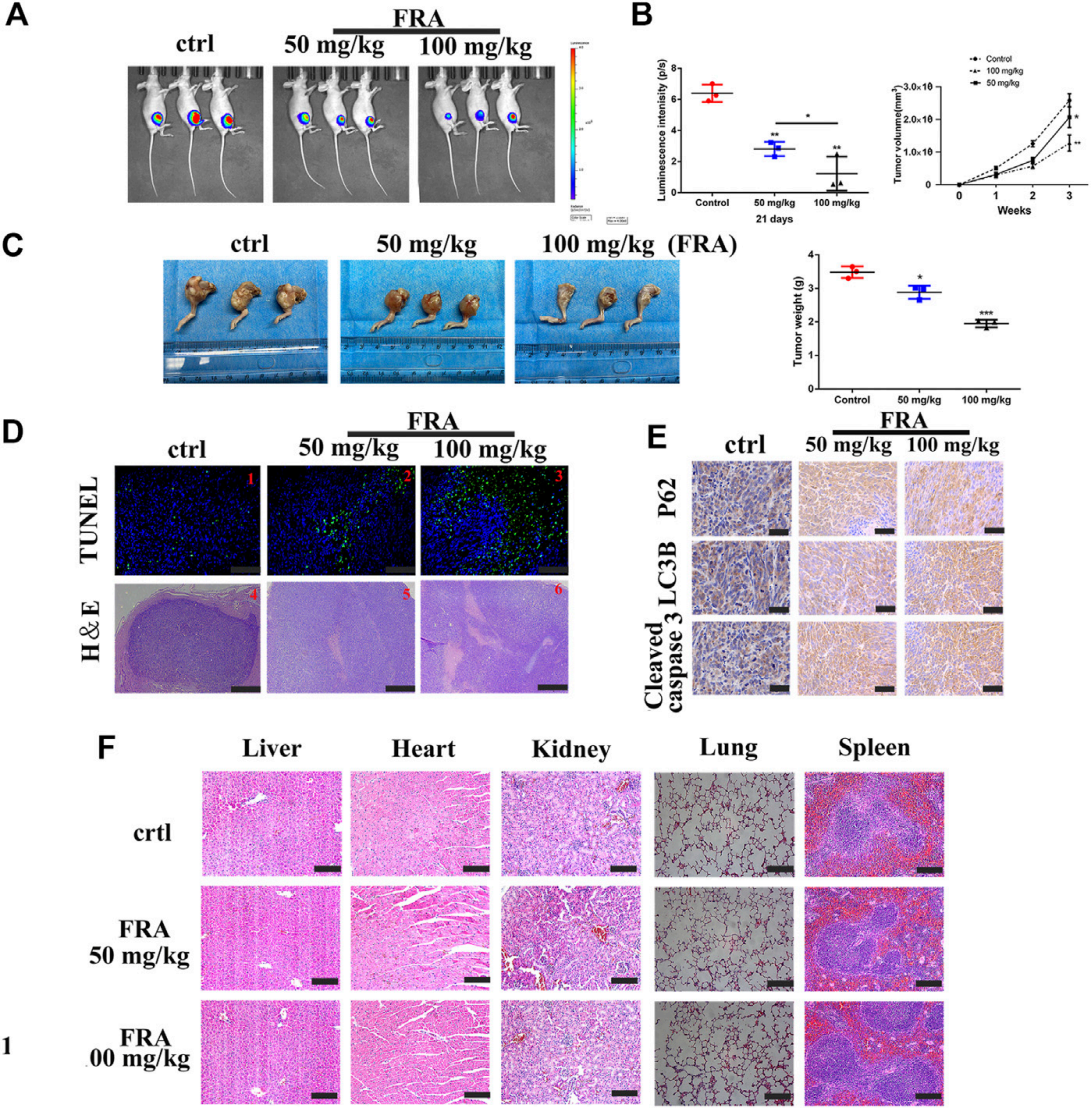
FRA hinders osteosarcoma growth *in vivo*. **(A)** After 21 days of FRA treatment, luciferase intensity of tumor was measured and calculated by an *in vivo* imaging system. **(B)** The luciferase intensity of tumor was analyzed. The curves of tumor volume showed a significant difference among the groups after 3 weeks administration of FRA. **(C)** The photographs show that there was a significant difference in tumor weights between the three groups after 21 days. **(D)** TUNEL assays were used to measure the apoptotic status of tumor tissues. FRA promoted extensive tumor cell necrosis was proved by H&E staining. **(E)** The levels of LC3B, P62 and cleaved-caspase 3 were further examined by immunohistochemistry. **(F)** The vital organs of mice (including the heart, liver, spleen, lungs and kidneys) did not show significant toxicity throughout the experimental. Error bar = mean ± SEM. Magnification, ×200 (D1–3), ×40 (D4-6), ×400 **(E)**. Scale bar, 100 μm (D1-3), 500 μm (D4–6), 50 μm **(E)**. Error bar = mean ± SD. *p < 0.05, **p < 0.01.

The last part of this research was to explore the anti-tumor effect of FRA *in vivo*. We followed the conventional modeling method, that is, injecting the HOS cell, stably transfected with luciferase, into the tibia marrow cavity of nude mice. As shown in Figure 5A, the level of fluorescence intensity meant the size of the tumor in mice. Compared with the control group, the fluorescence intensity of the FRA treatment group was significantly weaker, and the tumor intensity of the high-dose group was lower than that of the low-dose group. The pictures of Figures 5B,C intuitively showed that after 21 days of FRA treatment, there were differences in tumor size and weight between the FRA-treatment group and the control group, as well as the high and low dose groups. These data indicated that FRA exhibited a good anti-tumor ability *in vivo*. The subsequent histopathological examinations include TUNEL, H&E staining and immunohistochemistry. TUNEL and H&E confirmed that there were more dead cells in the tumor sections of the FRA treatment group (Figure 5D). As shown in Figure 5E, immunohistochemical quantitative analysis showed that the expression levels of cleaved-caspase 3, LC3B, and P62 in the FRA group were higher than those in the control group, which indicated that FRA also induced autophagy and apoptosis of osteosarcoma *in vivo.* In addition, as shown in Figure 5F, the vital organs of mice (including the heart, liver, spleen, lungs and kidneys) did not show significant toxicity throughout the experimental. All in all, FRA inhibits the growth of osteosarcoma *in vivo*, induces autophagy pathway and promotes apoptosis.

## Discussion

Osteosarcoma is one of the most common primary malignant bone tumors in the world, which mainly occurs in children and adolescents (Simpson et al., 2017). Different subtypes or grades of cancer cells lead to different treatment options for patients with osteosarcoma. For patients with low or moderate grade osteosarcoma, extensive resection is the main treatment. However, patients with high-grade osteosarcoma often require multiple treatments, including surgery, radiation therapy or chemotherapy (Bishop et al., 2016). Although surgery and chemotherapy have increased the disease-free survival rate to more than 60%, many patients still suffer from poor clinical outcomes, such as high recurrence rate, low survival rate of patients after cancer metastasis (Meazza and Scanagatta, 2016). Although many large-scale clinical trials have been conducted to adjust the dose of chemotherapy or combined with immunotherapy to improve the prognosis, the survival rate of patients with osteosarcoma metastasis has not been significantly improved (Kansara et al., 2014). Considering that traditional Chinese medicine has demonstrated various anti-tumor effects and mechanisms in the past few thousand years, we explored the anti-osteosarcoma effects of FRA in this study. Our study found that FRA can inhibit the proliferation and migration of osteosarcoma cell lines HOS and MG63 *in vitro.*

Fraxinellone (FRA) is a degradable limonin isolated from the root bark of the *Dictamnus* plant, which has relatively strong insecticidal activity (Bailly and Vergoten, 2020). In addition, the natural product also has anti-inflammatory and immunomodulatory effects (Lei et al., 2020). Recent studies have shown that it can induce the downregulation of the TGFβ signal transduction pathway to inactivate cancer-related fibroblasts, and cause a decrease in the number of M2 macrophages *in vivo* and *in vitro*, thereby inducing remodeling of the tumor microenvironment (Hou et al., 2018). In this research, we want to explore whether it has a direct killing effect on tumor cells. Recently, studies on the premature senescence model induced by H_2_O_2_ have explored the effect of FRA on senescence inhibition, and proved that the activation of AMPK signaling pathway and autophagy play a role in this process. And it is proposed that FRA can be considered as a new drug with potential anti-aging drugs (Kim et al., 2009).

Autophagy is an extremely complex biological behavior, and its role in tumors such as osteosarcoma is also contradictory (Ravanan et al., 2017). In the initial stage of tumorigenesis, autophagy is proposed to have anti-tumor effects. The specific mechanism is to inhibit the occurrence of chromosomal mutations, reduce oxidative stress, and induce autophagy cell death to prevent distant metastasis. In the later stages of tumorigenesis, autophagy maintains tumor cell homeostasis by various means to promote cancer progression or tumor metastasis to act as a tumor activator (Shibutani et al., 2015; Huang et al., 2018). In the current study, we investigated the potential role of FRA in the regulation of autophagy and found that the application of FRA can enhance autophagy in osteosarcoma cells. In addition to autophagy, FRA also significantly promotes the apoptosis of osteosarcoma cells, and this effect is dose-dependent. Further studies have shown that the use of chloroquine to inhibit autophagy activation can promote cell apoptosis. The presence of autophagy inhibitors can weaken the pro-apoptotic effect of FRA. The pro-autophagy effect of FRA we inferred is consistent with previous research conclusions of others (Han et al., 2018). Moreover, excessive autophagy can promote tumor cell apoptosis is also a generally accepted research conclusion, which may be because a single protein that plays an important role in autophagy may have other roles in pro-apoptotic signal transduction (Mariño et al., 2014). At present, the potential of autophagy regulation in the treatment of tumors and other diseases is confusing due to the complex interaction with apoptosis. A comprehensive understanding of the interaction between autophagy and apoptosis and the molecular mechanism is the main challenge for current research (Maiuri et al., 2007; Kaminskyy and Zhivotovsky, 2014). Unfortunately, in our research, we did not find an important protein target between the two after the application of FRA. Moreover, considering the cost and time cost, we did not explore the important signal pathways between the two, such as P53/DAPK/BH3 only proteins.

Previous reports have confirmed the anti-inflammatory and neuroprotective effects of FRA *in vivo* (Hou et al., 2018; Bailly and Vergoten, 2020; Lei et al., 2020). However, there are few studies on the anti-tumor effects of FRA. We described the autophagy and apoptosis regulation effects of FRA on osteosarcoma cells for the first time, and confirmed that FRA can inhibit tumor growth *in vivo*. Based on the results of previous studies (Xing et al., 2018), we chose 30 mg/kg and 100 mg/kg for *in vivo* research. However, the results suggested that the dose of 30 mg/kg did not exhibit a significant tumor suppressor effect, so we considered 50 mg/kg as the low dose and 100 mg/kg as the high dose. The tumor size and weight of the FRA treatment group were significantly lower than those of the control group, and the tumors of the high-dose group did not protrude from the muscle layer of the mice. In addition, during our experiment, no side effects of using FRA (loss of appetite, blood in the stool, lethargy, etc.) were observed, and no obvious necrosis was observed in the HE staining of important organ sections, which indicates that FRA is safe. Unfortunately, at the end of the experiment, we did not find any metastases in the lungs of any mouse (referring to osteosarcoma is the most prone to lung metastasis, so we focus on the lungs). We found that the presence of FRA can enhance the sensitivity of mice to the chemotherapy drug cisplatin, thereby enhancing its anti-tumor effect. And this effect may be related to PD-L1 and macrophages. Our research lays the first step for the follow-up anti-tumor application of FRA, and also provides theoretical support for other research. This result makes it impossible for us to resolve whether FRA It can inhibit the metastasis of osteosarcoma. These results indicate that FRA may be a promising anti-osteosarcoma drug, because FRA can inhibit the growth of osteosarcoma and has biological safety.

In short, FRA can inhibit the proliferation and migration of osteosarcoma, and also promote apoptosis by inducing autophagic flux. In addition, FRA shows good anti-tumor activity and has no obvious side effects. Our results provide a potential direction for traditional Chinese medicine in the treatment of osteosarcoma.

## Data Availability

The original contributions presented in the study are included in the article/Supplementary Material, further inquiries can be directed to the corresponding author.

## Abbreviations

ATG, autophagy-related genes; DMEM, dulbecco’s modified eagle’s medium; FBS, fetal bovine serum; FRA, fraxinellone; HOS, human osteosarcoma cell lines MNNG/HOS; TEM, transmission electron microscopy.
